# Experimental viral spillover across 25 million year gap in Rodentia reveals limited viral transmission and purifying selection of a picornavirus

**DOI:** 10.1128/mbio.01650-24

**Published:** 2024-09-06

**Authors:** Frances K. Shepherd, Shanley N. Roach, Autumn E. Sanders, Yanan Liu, Dira S. Putri, Rong Li, Nathan Merrill, Mark J. Pierson, Sergei V. Kotenko, Zhongde Wang, Ryan A. Langlois

**Affiliations:** 1 Department of Microbiology and Immunology, University of Minnesota, Minneapolis, Minnesota, USA; 2 Department of Animal, Dairy, and Veterinary Sciences, Utah State University, Logan, Utah, USA; 3 Department of Lab Medicine and Pathology, University of Minnesota, Minneapolis, Minnesota, USA; 4 Department of Microbiology, Biochemistry and Molecular Genetics, Rutgers New Jersey Medical School, Newark, New Jersey, USA; Washington University in St. Louis, St. Louis, Missouri, USA

**Keywords:** Zoonosis, virome, intrahost evolution

## Abstract

When a virus crosses from one host species to another, the consequences can be devastating. However, animal models to empirically evaluate cross-species transmission can fail to recapitulate natural transmission routes, physiologically relevant doses of pathogens, and population structures of naturally circulating viruses. Here, we present a new model of cross-species transmission where deer mice (*Peromyscus maniculatus*) are exposed to the natural virome of pet store mice (*Mus musculus*). Using RNA sequencing, we tracked viral transmission via fecal–oral routes and found the evidence of transmission of murine astroviruses, coronaviruses, and picornaviruses. Deep sequencing of murine kobuvirus revealed tight bottlenecks during transmission and purifying selection that leaves limited diversity present after transmission from *Mus* to *Peromyscus*. This work provides a structure for studying viral bottlenecks across species while keeping natural variation of viral populations intact and a high resolution look at within-host dynamics that occur during the initial stages of cross-species viral transmission.

**IMPORTANCE:**

Viral spillover events can have devastating public health consequences. Tracking cross-species transmission in real-time and evaluating viral evolution during the initial spillover event are useful for understanding how viruses adapt to new hosts. Using our new animal model and next generation sequencing, we develop a framework for understanding intrahost viral evolution and bottleneck events, which are very difficult to study in natural transmission settings.

## OBSERVATION

The biodiversity of viruses is immense, with over 1.6 million that can infect mammals and birds (1). While many viruses are host-species restricted, given the right circumstances viruses can overcome structural and immune barriers to infect a new host species. Over the past century, there have been several significant viral zoonotic events including 1918 influenza, human immunodeficiency virus (HIV), Ebola, Zika, and the SARS-CoV-2 pandemic (2, 3). Despite the potentially devastating consequences of cross-species transmission, spillover events are difficult to study and predict. There is a need for experimental systems that model natural viral transmission across species and evaluate barriers to infection, including the innate immune system.

Experimental approaches to study cross-species transmission include sequence-based or serological surveillance of hosts for the evidence of viral infections and reductionist experimental models directly infecting animals in laboratory settings. Although large-scale sampling is beneficial for identifying potential new virus-host interactions, these data often lack access to the entire transmission chain including the specific infectious reservoir and the recipient host species. They can also fail to capture transient infections, rare events, or dead-end transmissions. Reductionist infection models can model the entire transmission chain and make use of knockout animals or blocking antibodies to test the impact of innate immune barriers on viral replication. However, lab-grown virus stocks can alter virus diversity (4) and potential transmission barriers and bottlenecks could be masked when natural routes of transmission, and physiological doses are not used.

To improve upon approaches for studying cross-species transmission, we developed a model system to evaluate natural virus transmission using mice from pet stores and exposing laboratory animals to the microorganisms they carry (5). “Dirty” pet store mice are an excellent model reservoir host as they harbor a rich virome that reflects the natural biological diversity of viruses. Here, we use deer mice (*Peromyscus maniculatus*) as the recipient host to empirically evaluate viral transmission across a ~25 million year evolutionary gap (6). We also generated STAT2^−/^*^−^ Peromyscus* to evaluate interferon (IFN) signaling as a barrier to cross-species viral transmission.

**FIG 1:**
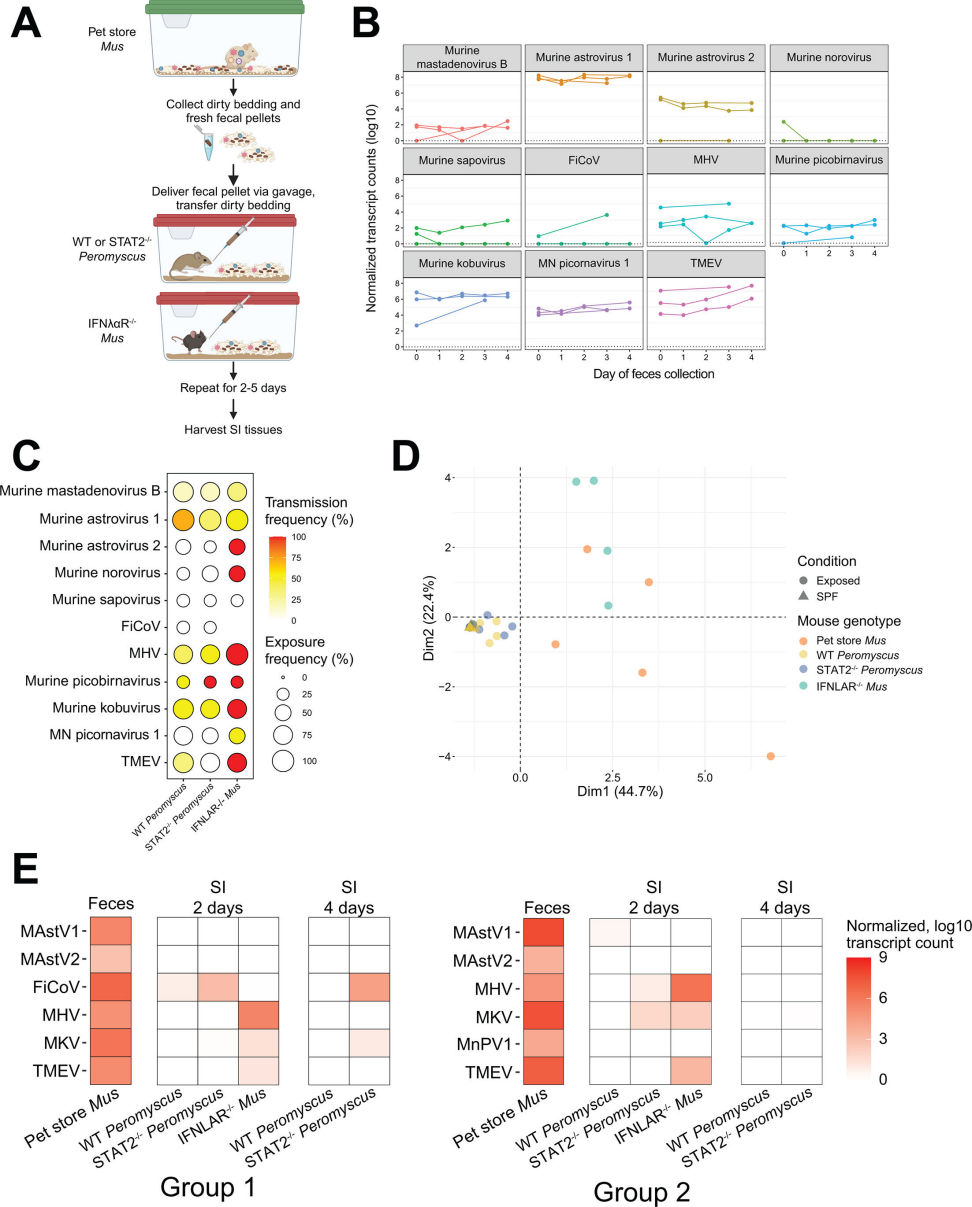
Cross-species transmission model of murine viruses. (A) Schematic of cross-species transmission experiment between pet store *Mus musculus* and *Peromyscus maniculatis. M. musculus* lacking IFNαλ receptors were included as a positive control for the transmission of infectious material. (B) Dynamics of virus species shedding in pet store mouse reservoir as measured via bulk RNAseq. Each line represents data from an individual pet store mouse sampled over time. (C) Exposure and transmission frequencies of viruses to new hosts. The percent of *Peromyscus* or IFNαλ^−/−^ *Mus* exposed to feces from a virus-positive pet store mouse (exposure frequency/dot size) and the percent of animals with subsequent detection of virus (transmission frequency/dot coloration) were calculated (*N* = 7 WT *Peromyscus*, *N* = 8 STAT2^−/−^ *Peromyscus*, and *N* = 4 IFNαλ^−/−^ *Mus*). (D) Principal component analysis (PCA) of virome composition between exposed animals and pet store mice (circles). Specific pathogen free (SPF) *Peromyscus* were sequenced as controls and plotted for comparison (triangles). PCA was performed based on read counts summarized at the virus family level. (E) Levels of virus species, measured by bulk RNAseq-normalized read counts, after either 2 or 4 days of exposure to pet store mice virome. Panel B represents shedding data from three pet store mice. Panels C and D represents data from five pet store mice transmission experiments. Panels E represents data from two additional pet store mice transmission experiments, separate from Panels C and D. MAstV1, murine astrovirus 1; MAstV2, murine astrovirus 2; FiCoV, Fievel mouse coronavirus; MHV, murine hepatitis virus; MKV, murine kobuvirus; MnPV1, Minnesota picornavirus 1; TMEV, Theiler’s murine encephalitis virus.

We exposed wild type (WT) and STAT2^−/−^ *Peromyscus* to the bedding and fecal contents of pet store mice daily for 3 or 5 days and harvested small intestine (SI) 24 h after the final dose (Fig. 1A). We used RNAseq to examine the diversity of viruses shed in pet store mouse feces and found up to 21 virus species with varying levels of abundance (Fig. 1B). We found a *Picornaviridae* contig with highest BLAST identity (86%) to an unclassified rodent picornavirus identified in China (7). We named this new strain Minnesota rodent picornavirus 1 (MnPV1).

We evaluated the SI of the exposed animals for viral RNA as the evidence of potential cross-species virus transmission (Fig. 1C). As a positive control for transmission, we assessed transmission to *M. musculus* deficient in IFN-a and -l receptors (IFNαλR^−/−^). Despite the large diversity of viruses in pet store mice, we found only limited potential cross-species transmission of murine astrovirus 1 (MAstV1), murine hepatitis virus (MHV), and murine kobuvirus (MKV) (Fig. 1C). Principal component analysis comparing SI viral reads at the family level showed that *Peromyscus* exposed to pet store mouse viruses have similar viromes to SPF *Peromyscus*, indicating host restriction of many murine viruses, even in the absence of STAT2 signaling (Fig. 1D). In contrast, IFNαλR^−/−^ *Mus* had diverse viromes after exposure to pet store viruses. We note that virus found in the SI via RNAseq could represent *bona fide* cross-species transmission or residual virus from the reservoir simply passing through the gastrointestinal tract. In pet store mice with high levels of murine astrovirus 2 (MAstV2) shed in feces, we detected no MAstV2 reads in the exposed deer mice. Given the environmental stability of astroviruses (8–11), this suggests the virus detected via RNAseq in deer mice is not a result of an ambient fecal material passing through the gut and that virus is cleared in deer mice if replication does not occur.

To determine whether the 5-day exposure timeline allowed us to fully examine viral transmission, we evaluated animals exposed to pet store fecal material for 2 or 4 days. After the final exposure, we allowed an additional 24 h of no exposure to allow for clearance of potential non-replicating virus. In this context, we detected viral reads of MHV, MKV, and Fievel mouse coronavirus (FiCoV; an alphacoronavirus) as early as 2 days (Fig. 1E). Interestingly, many viruses were not detected in the 4-day exposure group, suggesting that viruses detected in earlier timepoints but absent later may have undergone dead-end transmission.

**FIG 2:**
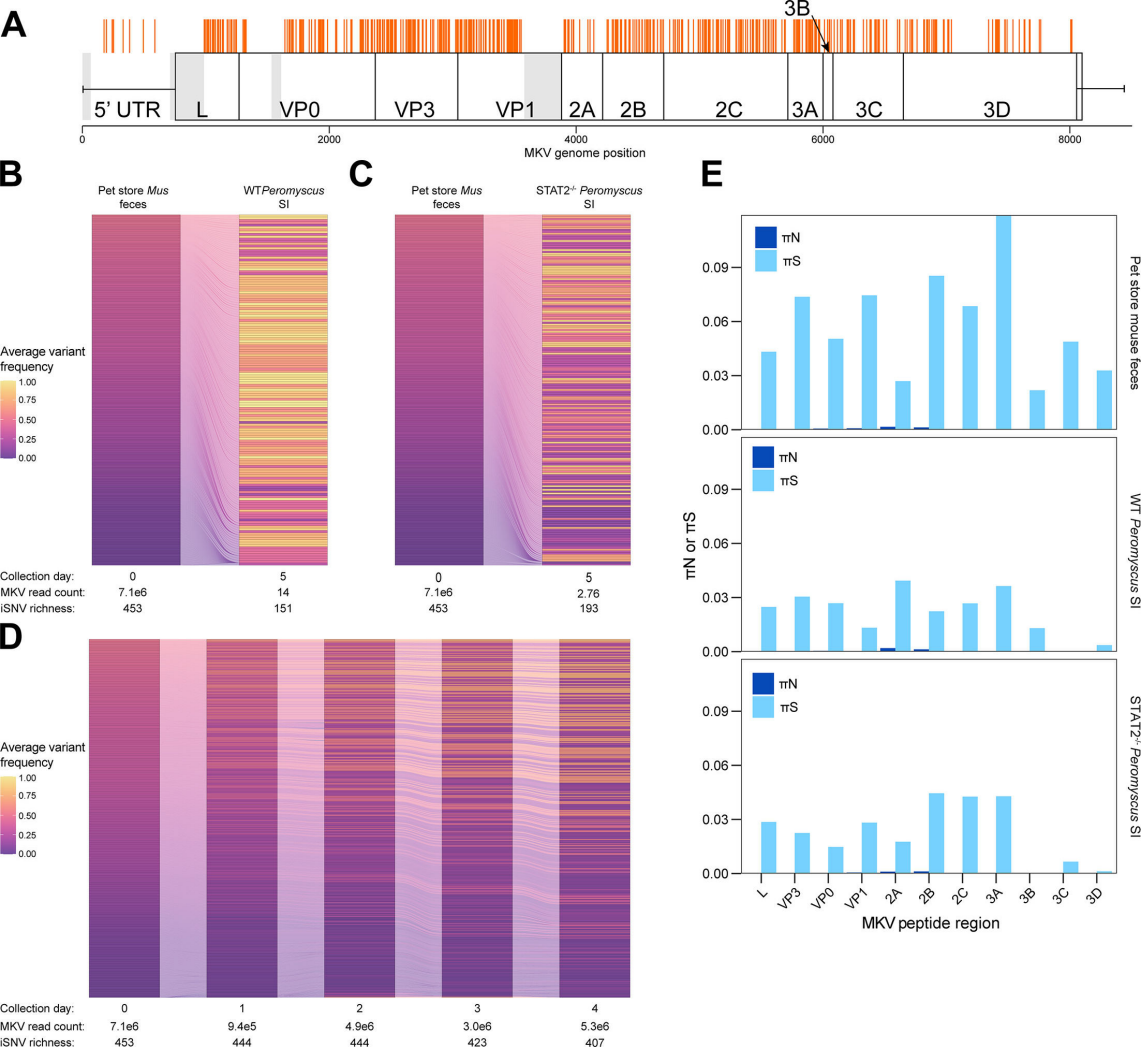
Changes in MKV iSNV frequencies during transmission. (A) iSNV locations in the initial fecal transmission. Orange represents positions of iSNVs in the MKV genome. Gray represents genome sections where primer amplification failed. (B and C) Changes in iSNV frequencies from the viral population in the initial pet store inoculum (left stratum in each panel) compared to *Peromyscus* SI at the day 5 endpoint (right stratum in each panel) of inoculated WT deer mouse (B) and STAT2^−/−^ deer mouse (C). Each row in the alluvial plot represents an iSNV, colored by its frequency in the viral population. Numbers below the stratum refer to normalized MKV read counts from RNAseq and iSNV richness. (D) Changes in viral iSNVs shed each day in the pet store mouse feces. All iSNVs were called against the consensus sequence generated from the initial viral fecal inoculum. Numbers below the stratum refer to normalized MKV read counts from RNAseq and iSNV richness. (E) Rates of synonymous mutations per synonymous site (πS) and nonsynonymous mutations per nonsynonymous site (πN) in MKV. Bars represent the mean rates for each peptide region across the two independent sequencing experiments. All variants were called against the MKV consensus sequence from the pet store mouse day 0 feces.

Despite the overall limited detection of murine viruses during cross-species exposure*,* MKV was one of the most frequently detected viruses in *Peromyscus*. Therefore, we used amplicon deep sequencing of MKV to characterize the evolution of viral variants during transmission to *Peromyscus*. We deep sequenced pet store mouse feces on day 0–day 4, and in SI tissues of *Peromyscus* at the experimental endpoint and called intrahost single nucleotide variants (iSNVs) using the day 0 feces as a consensus sequence. Pet store mouse feces contained a diverse range of iSNVs distributed across the MKV genome (Fig. 2A) at a range of sub-consensus frequencies (Fig. 2B and C, left columns). The iSNVs in the feces remained stable over time, with very few examples of iSNVs being lost or *de novo* variants arising in the reservoir (Fig. 2D). In contrast, when we compared the iSNV frequencies in *Peromyscus* SI to the day 0 fecal material, we found a lower richness of MKV iSNVs in the SI tissue on day 5 in the exposed animals (Fig. 2B and C, right columns) with a 66.7% and 59.4% reduction in iSNV richness in WT and STAT2^−/−^ *Peromyscus*, respectively. Surprisingly, there were more MKV reads in WT *Peromyscus* compared to STAT2^−/−^ and an increase in iSNV frequencies in the WT animal (Fig. 2B) while STAT2^−/−^ iSNVs remained at lower frequencies. Synonymous mutation rates (πS) far exceeded nonsynonymous mutation rates (πN) for all the regions of the MKV genome (Fig. 2E), indicating purifying selection occurred in MKV. Nonsynonymous changes were extremely rare, and the only two nonsynonymous changes that occurred in *Peromyscus* (A613G and P1075A in the 2A and 2B peptide regions, respectively) were also present in the pet store mouse. Together, this suggests the MKV population in *Peromyscus* arises from purifying selection and a narrow bottleneck during transmission from the reservoir.

Zoonosis and other spillover events can occur between closely related animals or between distantly related species. For example, simian immunodeficiency virus (SIV) jumped a 6 million-year-gap from chimpanzees to humans (12). Using our model, we found that viruses from multiple different families can sporadically cross an even larger evolutionary distance barrier. However, the low viral reads suggests that many of the viruses detected in *Peromyscus* after transmission from *Mus* likely represent dead-end transmission events. These events are often difficult to detect, as dead-end infections may not lead to significant clinical signs. This model, however, offers an exciting opportunity to evaluate how viruses evolve during dead-end transmission under more natural transmission contexts. Future work should focus on understanding what factors are necessary to help viruses overcome dead-ends and establish productive infections.

The loss of viral iSNVs in *Peromyscus* suggests that MKV undergoes a tight bottlenecking event during transmission between host species. Tight bottlenecks have also been observed in other transmission contexts and viral species, including influenza A virus (IAV) among ferrets (13) and humans (14); cross-species IAV transmission between chicken and humans (15); coxsackievirus transmission among mice (16); and SARS-CoV-2 transmission among cats (17). Importantly, these bottlenecks are observed even when viruses are transmitted within a single host species. We did not detect the evidence of positive selection or accumulation of nonsynonymous changes in MKV that would indicate adaptation to a new host species. Without further adaptation, onward transmission of MKV may be limited. Our findings align with evidence for purifying selection within avian IAV sequences from duck and human (15). Longer experimental time scales may be necessary to detect nonsynonymous changes, though the ability to undergo even a few initial rounds of replication suggests MKV could be equipped for sustained cross-species transmission given the right conditions.

In summary, we found low-frequency transmission of murine viruses to *Peromyscus* hosts and strong purifying selection of MKV during the initial rounds of replication. This highly tractable model system uniquely combines the ability to perturb the immune system of infected hosts while keeping the natural genetic variation of viral populations intact. Thus, we can model the most complex aspects of viral transmission, such as viral quasispecies dynamics or multi-strain transmission, which are often impossible to directly study in hosts in their natural environments. This deer mouse cross-species transmission model can be applied more broadly to investigate co-transmission of viruses, the impact of the microbiome on viral transmission, and the intrahost evolution of other murine viruses during host jumps.

## Data Availability

All sequencing data from this study are publicly available under NCBI BioProject accession number PRJNA1133789. Minnesota rodent picornavirus 1 whole genome contigs are deposited in GenBank under accession numbers PQ110019-PQ110028. Murine kobuvirus whole genome contigs used for amplicon primer design are deposited in GenBank under accession numbers PQ110014-PQ110018. Code used to analyze these data are publicly available at https://github.com/langloislab/shepherd_et_al_deer_mouse.

## SUPPLEMENTAL MATERIAL

Supplemental material mbio.01650-24-s0001.docx Supplemental methods.

TABLE S1 mbio.01650-24-s0002.xlsx Experimental design for Fig. 1D and E.

TABLE S2 mbio.01650-24-s0003.xlsx Murine kobuvirus amplicon sequencing primers.

ASM does not own the copyrights to Supplemental Material that may be linked to, or accessed through, an article. The authors have granted ASM a non-exclusive, world-wide license to publish the Supplemental Material files. Please contact the corresponding author directly for reuse.
