# Supplementary material for: Experimental viral spillover across 25 million year gap in Rodentia reveals limited viral transmission and purifying selection of a picornavirus
Source: mBio. 2024 Sep 6;15(10):e01650-24. doi: 10.1128/mbio.01650-24 (PMC11481857; doi:10.1128/mbio.01650-24)
Supplement: Supplemental material — Supplemental methods. [file mbio.01650-24-s0001.docx]

**Shepherd, et al.**

**Supplementary methods**

**Animal experiments**

All animal experiments were performed in a BSL3 facility to protect specific-pathogen free (SPF) mouse colonies. Pet store mice were purchased from pet stores in the Twin Cities region of Minnesota. Wild-type *Peromyscus maniculatus* (deer mice) were purchased from the University of South Carolina Peromyscus Genetic Stock Center. The *STAT2*^-/-^ *Peromyscus maniculatus* model was generated with CRISPR/Cas9-mediated gene editing developed in the Wang laboratory (Liu Y et al., manuscript in preparation) at Utah State University. Wild-type deer mice used for generating the *STAT2*^-/-^ model were produced from a breeding colony established in house at Utah State University from animals provided as gifts by Dr. Tony Schountz at Colorado State University (1) . B6.IL- 28RA^-/-^Ifnar1^-/-^ (IFNαλR^-/-^) were generated as described previously (2).

For transmission experiments, all animals were housed individually. We exposed SPF *Peromyscus* or *Mus* animals to the virome of pet store mice via oral gavage of feces and dirty bedding transfer. For gavage, fresh fecal pellets were collected from pet store mice and vortexed in 1 mL of PBS to create a fecal slurry. 100 uL of the slurry was delivered via gavage to each animal. Soiled bedding was collected from the cage of the pet store mouse and added to the cage of the *Peromyscus* or *Mus* animal. Fresh bedding was added to pet store mouse’s cage after transferring out dirty bedding. We evaluated viral transmission using multiple experimental set ups (Table S1). In the first set up, animals were gavaged for 3 full days and dirty bedding was transferred for 2 days. All animals were harvested on the 4^th^ day. In the second set up, animals were gavaged for 5 days and dirty bedding was transferred for 4 days. All animals were harvested on the 6^th^ day. A third set up was used to compare time of exposure and viral transmission within a single experimental group (Fig. 1E). Here, a single transmission experiment consisted of a 2-day exposure group with one WT *Peromyscus*, one STAT2^-/-^ *Peromyscus*, and one IFNLAR^-/-^ *Mus*, and a 4-day exposure group with one WT *Peromyscus* and one STAT2^-/-^ *Peromyscus.* The 2-day exposure group was gavaged for 2 days and received dirty bedding on one day. The animals were harvested on the 4^th^ day. The 4-day exposure group was gavaged for 4 days and received dirty bedding for 3 days. The animals were harvested on the 5^th^ day. See Table S1 for experimental group details.

**Tissue collection**

Pet store mouse feces were collected and flash frozen on dry ice during each day of the experiment. RNA was extracted from feces using the PowerFecal DNA/RNA kit (Qiagen). On the final day of the experiments, exposed animals and the pet store mouse were euthanized and small intestines, liver, and lung tissues were collected. Tissues were homogenized in Buffer RLT Plus (Qiagen) with 2-mercaptoethanonl (10 uL/1mL) and Reagent DX (0.5% v/v, Qiagen) using a GentleMacs M tube (Miltenyi Biotec). RNA was extracted with the AllPrep DNA/RNA Mini kit (Qiagen).

**Sequencing**

Feces, small intestine, liver and lung tissues were all used for RNA sequencing. PolyA-enriched cDNA libraries were prepared using the Kapa Hyper Stranded mRNA library prep kit (Roche). Sequencing was performed using NovaSeq 6000 or NovaSeq X Plus, with 150 bp paired end reads.

For deep sequencing, only feces and small intestinal tissues were used. Amplicons spanning the MKV whole genome were generated in duplicate from RNA samples. Primers were designed to amplify overlapping regions of approximately 500bp with PrimalScheme (3) based on *de novo* assembled whole genome contigs from bulk RNAseq data (deposited under Genbank accession numbers PQ110014-PQ110018) (Table S2). All primers are listed in Table S2. cDNA was generated using SuperScript II reverse transcriptase with random hexamer priming (Invitrogen). Tiled amplicons were generated from cDNA using Q5 High Fidelity DNA polymerase (New England Biolabs) and a final concentration of 0.02 uM per primer in each reaction mastermix. Bands of the correct size were confirmed by gel electrophoresis and purified using the NucleoSpin Gel and PCR Clean-up kit (Macherey-Nagel). Libraries were prepared with the Kapa Hyper Prep kit (Roche) and paired-end 250bp reads were generated using the MiSeq v3 platform (Illumina). Two independent amplicons were generated from RNA for each sample.

**Data Analysis**

RNAseq data depicted in figure 1 were generated by first mapping reads to the appropriate host genome (either *Mus musculus* or *Peromyscus maniculatus*) using STAR version 2.7.1a (4)**. Unmapped reads from all animals within an exposure group (and any paired, unexposed SPF deer mice included as negative controls) were concatenated and *de novo* assembled with Trinity version 2.12.0 (5). Contigs were assigned taxonomic lineages using BLASTn (6). The set of contigs per experiment were used as an index for Salmon (7) to estimate transcript counts per sample. Counts were summarized at either the species or family level and normalized with DESeq2 (8). Normalized counts were log transformed and graphed using ggplot2 (9). Downstream analysis and graphing only included fecal and small intestine reads to focus on fecal-oral transmission of viruses. Read counts were additionally averaged across all negative control SPF animals and subtracted from read counts of exposed animals to correct for any potential contamination.**

**Exposure rates of virus species were determined by finding the proportion of animals that were in a group with a pet store mouse with virus detected in the small intestinal tissue. Within exposed animals, transmission rates were calculated as the proportion of animals with subsequent viral detection in the small intestine. Principal components analysis was performed using normalized read counts at the family level with the stats package in R (10).**

Amplicon deep sequencing data was analyzed using iVar (11)**.** A MKV consensus sequence was generated from the pet store mouse feces on the first day of the experiment (day 0) by pooling reads from the two sequencing replicates and mapping them to **a *de novo* assembled whole genome contig from one of the transmission experiments (deposited under accession number** PQ110014**). iSNVs were called by mapping** reads to the generated pet store mouse consensus. Reads generated from primers with mismatches to the generated consensus sequence were masked from further analysis due to mismatches potentially causing incorrect variant frequency calculations (11). iSNVS were called at regions with a minimum quality threshold of 30, sequencing depth of 100X, and a frequency of 3%. Finally, any iSNVS present in only one sequencing replicate were dropped. The remaining iSNV frequencies were averaged between the two replicates for further analysis.

To evaluate selection pressure within MKV populations, average number of nonsynonymous changes per nonsynonymous site (πN) and synonymous changes per synonymous site (πS) were calculated using SNPGenie (12).

**Supplemental References**

1. Schountz T, Green R, Davenport B, Buniger A, Richens T, Root JJ, Davidson F, Calisher CH, Beaty BJ. 2004. Cloning and characterization of deer mouse (Peromyscus maniculatus) cytokine and chemokine cDNAs. BMC Immunol 5:1.

2. Lin J-D, Feng N, Sen A, Balan M, Tseng H-C, McElrath C, Smirnov SV, Peng J, Yasukawa LL, Durbin RK, Durbin JE, Greenberg HB, Kotenko SV. 2016. Distinct Roles of Type I and Type III Interferons in Intestinal Immunity to Homologous and Heterologous Rotavirus Infections. PLoS Pathog 12:e1005600.

3. Quick J, Grubaugh ND, Pullan ST, Claro IM, Smith AD, Gangavarapu K, Oliveira G, Robles-Sikisaka R, Rogers TF, Beutler NA, Burton DR, Lewis-Ximenez LL, de Jesus JG, Giovanetti M, Hill SC, Black A, Bedford T, Carroll MW, Nunes M, Alcantara LC, Sabino EC, Baylis SA, Faria NR, Loose M, Simpson JT, Pybus OG, Andersen KG, Loman NJ. 2017. Multiplex PCR method for MinION and Illumina sequencing of Zika and other virus genomes directly from clinical samples. 6. Nat Protoc 12:1261–1276.

4. Dobin A, Davis CA, Schlesinger F, Drenkow J, Zaleski C, Jha S, Batut P, Chaisson M, Gingeras TR. 2013. STAR: ultrafast universal RNA-seq aligner. Bioinformatics 29:15–21.

5. Grabherr MG, Haas BJ, Yassour M, Levin JZ, Thompson DA, Amit I, Adiconis X, Fan L, Raychowdhury R, Zeng Q, Chen Z, Mauceli E, Hacohen N, Gnirke A, Rhind N, di Palma F, Birren BW, Nusbaum C, Lindblad-Toh K, Friedman N, Regev A. 2011. Trinity: reconstructing a full-length transcriptome without a genome from RNA-Seq data. Nat Biotechnol 29:644–652.

6. Chen Y, Ye W, Zhang Y, Xu Y. 2015. High speed BLASTN: an accelerated MegaBLAST search tool. Nucleic Acids Res 43:7762–7768.

7. Patro R, Duggal G, Love MI, Irizarry RA, Kingsford C. 2017. Salmon: fast and bias-aware quantification of transcript expression using dual-phase inference. Nat Methods 14:417–419.

8. Love MI, Huber W, Anders S. 2014. Moderated estimation of fold change and dispersion for RNA-seq data with DESeq2. Genome Biol 15:550.

9. Wickham H. 2016. ggplot2: Elegant Graphics for Data Analysis. Springer-Verlag New York. https://ggplot2.tidyverse.org.

10. R Core Team. 2018. R: A Language and Environment for Statistical Computing (3.5.2). R Foundation for Statistical Computing, Vienna, Austria.

11. Grubaugh ND, Gangavarapu K, Quick J, Matteson NL, Jesus JGD, Main BJ, Tan AL, Paul LM, Brackney DE, Grewal S, Gurfield N, Rompay KKAV, Isern S, Michael SF, Coffey LL, Loman NJ, Andersen KG. 2019. An amplicon-based sequencing framework for accurately measuring intrahost virus diversity using PrimalSeq and iVar. 1. Genome Biol 20:1–19.

12. Nelson CW, Moncla LH, Hughes AL. 2015. SNPGenie: estimating evolutionary parameters to detect natural selection using pooled next-generation sequencing data. Bioinformatics 31:3709–3711.
